# Factors affecting the relationship between psychological status and quality of life in COPD patients

**DOI:** 10.1186/1477-7525-8-108

**Published:** 2010-09-27

**Authors:** Eva Balcells, Joaquim Gea, Jaume Ferrer, Ignasi Serra, Mauricio Orozco-Levi, Jordi de Batlle, Esther Rodriguez, Marta Benet, David Donaire-González, Josep M Antó, Judith Garcia-Aymerich

**Affiliations:** 1 Servei de Pneumologia, Hospital del Mar-IMIM, Passeig Marítim 25-29, 08003 Barcelona, Spain; 2 Municipal Institute of Medical Research (IMIM-Hospital del Mar), Doctor Aiguader 88, 08003 Barcelona, Spain; 3 CIBER de Enfermedades Respiratorias (CIBERES), Recinte Hospital Joan March, Carretera Soller km 12, 07110 Bunyola, Spain; 4 Department of Experimental and Health Sciences, Universitat Pompeu Fabra, Doctor Aiguader 88, 08003 Barcelona, Spain; 5 Servei de Pneumologia, Hospital General Universitari Vall D'Hebron, Passeig Vall d'Hebron 119-129, 08035 Barcelona, Spain; 6 Centre for Research in Environmental Epidemiology (CREAL), Doctor Aiguader 88, 08003 Barcelona, Spain; 7 CIBER Epidemiología y Salud Pública (CIBERESP), Doctor Aiguader 88, 08003 Barcelona, Spain

## Abstract

**Background:**

This study aims to (i) evaluate the association between anxiety and depressive symptoms and health-related quality of life (HRQoL); and (ii) identify the effect modifiers of this relationship in patients with chronic obstructive pulmonary disease (COPD).

**Methods:**

A total of 337 clinically stable COPD patients answered the St. George's Respiratory Questionnaire (SGRQ) (assessing HRQoL) and the Hospital Anxiety and Depression Scale (HADS). Socio-demographic information, lung function, and other clinical data were collected.

**Results:**

Most patients (93%) were male; they had a mean (SD) age of 68 (9) years and mild to very severe COPD (post-bronchodilator FEV_1 _52 (16)% predicted). Multivariate analyses showed that anxiety, depression, or both conditions were associated with poor HRQoL (for all SGRQ domains). The association between anxiety and total HRQoL score was 6.7 points higher (indicating a worse HRQoL) in current workers than in retired individuals. Estimates for patients with "both anxiety and depression" were 5.8 points lower in stage I-II than in stage III-IV COPD, and 10.2 points higher in patients with other comorbidities than in those with only COPD.

**Conclusions:**

This study shows a significant association between anxiety, depression, or both conditions and impaired HRQoL. Clinically relevant factors affecting the magnitude of this association include work status, COPD severity, and the presence of comorbidities.

## Background

Health-related quality of life (HRQoL), defined as the degree to which a patient's health status affects his or her self-determined evaluation of satisfaction or quality of life [1], is an important prognostic factor in COPD patients. Poor HRQoL has been associated with a greater risk of hospitalisation [2] and mortality [3] in these patients. A large body of literature examines HRQoL correlates in COPD patients, and has particularly focused on factors such as pulmonary function impairment, exercise capacity, dyspnea, and the presence of comorbidities [4-7]. More recently, some reviews have highlighted the negative impact of psychological comorbidity on HRQoL in COPD patients [8,9]. Specifically, previous studies of large samples of COPD patients have found an association between psychological impairment (i.e., anxiety and/or depressive symptoms) and worse respiratory-specific HRQoL, independent of COPD severity [10-12]. An aspect poorly explored in previous research is the identification of effect modifiers of the relationship between psychological status and HRQoL. Only one earlier study has examined the differences in the association between anxiety and depression and HRQoL by severity groups [13]. It is currently not known whether socio-demographic factors or comorbidities modify the association between anxiety and depression and the HRQoL of COPD patients.

This study aimed to (1) evaluate the association between anxiety and depressive symptoms and HRQoL after adjusting for potential confounders and (2) identify the effect modifiers of this relationship. Authors hypothesised that the relationship between anxiety and/or depressive symptoms and HRQoL in COPD patients could be modified by certain socio-demographic and functional characteristics of the patients. The study included a large sample of stable COPD patients with mild to very severe disease, and had been designed as an ancillary study of the larger Phenotype and Course of COPD (PAC-COPD) project [14].

## Methods

### Design

This study used a cross-sectional analysis [14].

### Subjects

The study population included all subjects admitted to nine teaching hospitals in Spain for the first time for the exacerbation of COPD between January 2004 and March 2006. The diagnosis of COPD was confirmed by spirometry when the patient had reached clinical stability, at least three months after discharge; COPD was identified as a post-bronchodilator forced expiratory volume in one second to forced vital capacity ratio (FEV_1_/FVC) of less than or equal to 0.7 [15]. Of the 342 patients included in the PAC-COPD study, 337 (98.5%) had anxiety, depression, and HRQoL measures, and therefore were included in the present analysis. The study was approved by the ethics committees of all the participating hospitals and all patients gave their written informed consent. Additional details about the recruitment process have been previously published [16].

### Measurements

All measurements were completed when patients were clinically stable. Trained interviewers administered questionnaires to patients.

HRQoL was measured using the validated Spanish version of St. George's Respiratory Questionnaire (SGRQ) [17,18]. This is a disease-specific, health-related quality of life questionnaire consisting of 50 items with 76 weighted responses that cover 3 domains: Symptoms, Activity, and Impact. The "Symptoms" domain relates to the frequency and severity of respiratory symptoms, the "Activity" domain relates to activities that cause or are limited by breathlessness, and the "Impact" domain covers aspects of social function and psychological disturbances resulting from respiratory disease. A score is calculated for each domain and a total score is calculated. The scores range from 0 to 100 and a higher score indicates a worse HRQoL. The "minimally important difference" in a score that signifies a clinically significant change is considered to be four points [19].

Anxiety and depression were evaluated with the Spanish version of the Hospital Anxiety and Depression Scale (HADS) [20,21]. This is a validated screening questionnaire for psychiatric morbidity that is widely used in COPD patients [10,11,22]. This questionnaire has two 7-item subscales for anxiety and depression, respectively. The scores range from 0 to 21 for each subscale. A score of 8 or greater is used as the cut-off point for the identification of anxiety and depression, meaning that individuals with even mild symptoms are considered to have depression or anxiety in this study [20].

Limitations in activities of daily living were assessed by the Barthel index, which quantifies independence in self-care and mobility. The final score on the Barthel index ranges from 0, or complete dependence, to 100, or complete independence [23].

Other relevant information was also collected, including age, gender, marital status, level of education, socioeconomic status, work activity, smoking status, respiratory drug treatment, long-term oxygen therapy, dyspnea (measured by using the Modified Medical Research Council Scale, MMRC), nutritional status (body mass index (BMI, kg/m^2^)), the Charlson comorbidity index, lung function (forced spirometry and bronchodilator test, static lung volumes by whole-body plethysmography, diffusing capacity for carbon monoxide (DLco) and arterial blood gases), and six-minute walk test. Detailed information about the methods and sources of the questionnaires and the standardisation of the tests used in the PAC-COPD study has been previously published [14,16].

### Statistical analysis

Depending on the variable distribution, results are expressed as numbers and percentages, means and standard deviations (SD), or medians and 25th or 75th percentiles. The patients were classified into four stages of COPD severity according to the guidelines of the European Respiratory Society and the American Thoracic Society (ERS/ATS): mild COPD (FEV_1_≥80%); moderate COPD (FEV_1_≥50%, <80%); severe COPD (FEV_1_≥30%, <50%), and very severe COPD (FEV_1 _< 30%) [15]. SGRQ score, HADS score, and Barthel index were compared across the stages of COPD severity using ANOVA or Kruskal-Wallis (for quantitative variables) and Chi squared or Fisher's exact test (for qualitative variables). A multivariate linear regression model was built for each domain of SGRQ; anxiety and depression were the main explicative variables and potential confounders were identified from the literature, including age, gender, marital and socioeconomic status, level of education, work activity, smoking status, post-bronchodilator FEV_1 _(% of the predicted value), PaO_2_, PaCO_2_, DLco, residual volume/total lung capacity (RV/TLC), six-minute walking distance (6MWD), degree of dyspnea, BMI, Charlson comorbidities, and Barthel index. All covariates with a p-value <0.125 were entered into a multivariate regression model, and successively excluded if they were not associated with the outcome (p < 0.05) or its exclusion did not modify by 10% any of the remaining estimates. Finally, the most parsimonious model that still explains the data was built for each outcome. Goodness of fit was assessed through graphical study of the residuals with specific attention paid to whether they were randomly distributed around zero and exhibited small and constant variance [24]. All final multivariate models were stratified according to a list of *a priori *defined potential effect modifiers of the association between anxiety and depression and HRQoL; these potential modifiers included gender, age (categorised according to the median: less than 69 years or greater than or equal to 69 years), work status, smoking status, level of education, marital status, socioeconomic status, severity of COPD (according to the stages outlined by ERS/ATS [15]), and Charlson comorbidities. The group with "depression alone" was not included in the stratified analyses due to the small number of subjects (n = 16). To identify the variables independently associated with psychological impairment (anxiety, depression, or both), a multinomial (polytomous) logistic regression model was built, with neither anxiety nor depression as the reference group. The following confounders were tested and finally included in the final model if they were independently related to the outcome, or modified estimates for the remaining variables: age, gender, level of education, work status, marital status, dyspnea, FEV_1_, DLco, RV/TLC, PaO_2_, PaCO_2_, 6MWD, BMI and comorbidities. Analyses were performed using the statistical package SPSS 11.5 (2002, SPSS Inc., Chicago, United States).

## Results

Most patients (93%) were male and the mean age was 68 (SD: 9) years (range: 47 to 86 years); fifty-five patients (16%) were current workers. A wide spectrum of severity was found (mean (SD) post-bronchodilator FEV_1 _was 52 (16)% of the predicted value). More than 50% of the patients had two or more comorbidities, ranging from 1 (44% of patients) to 7 (1% of patients) comorbidities (Table 1). Table 2 shows that SGRQ scores increased (showing a worsening HRQoL) with increasing COPD severity. Only 9% of patients were dependent (Barthel index < 100), but this percentage increased up to 24% in those with very severe COPD. Symptoms of anxiety were more prevalent than depressive symptoms (27% and 14%, respectively); thirty patients (9%) had symptoms of both conditions. There were no significant differences in HADS scores across the stages of COPD.

**Table 1:** Socio-demographic and clinical characteristics of 337 patients with COPD.

	n = 337*
Males, n (%)	314 (93.2)

Age (years), m (SD)	67.9 (8.6)

Married, n (%)	272 (80.7)

Less than primary education, n (%)	139 (41.2)

Low socioeconomic status (IV-V)^†^, n (%)	255 (81.5)

Current workers, n (%)	55 (16.3)

Smoking status, n (%)	

Current smoker	108 (32.8)

Former smoker	219 (66.6)

Never smoked	2 (0.6)

FEV_1 _post-bronchodilator (% pred.), m (SD)	52.4 (16.2)

MMRC dyspnea scale, median (P_25_-P_75_)	2 (2-3)

RV/TLC (%), m (SD)	55.6 (10.1)

DLco (% pred.), m (SD)	65.2 (20.8)

PaO_2 _(mmHg), m (SD)	74.3 (10.7)

PaCO_2 _(mmHg), m (SD)	41.8 (5.3)

6MWD (m), median (P_25_-P_75_)	440.0 (390.0-508.4)

BMI (Kg/m^2^), m (SD)	28.2 (4.7)

Comorbidities: Charlson index ≥2^+^, n (%)	187 (55.8)

Any respiratory drug treatment, n (%)	288 (85.5)

Long-term oxygen therapy, n (%)	27 (8.2)

COPD, chronic obstructive pulmonary disease; FEV_1_, forced expiratory volume in one second; MMRC, Modified Medical Research Council; RV/TLC, residual volume/total lung capacity; DLco, diffusing capacity for carbon monoxide; PaO_2_, partial pressure of oxygen in arterial blood; PaCO_2_, partial pressure of carbon dioxide in arterial blood; 6MWD, 6-minute walking distance; BMI, body mass index.

* Some variables had missing values: 24 in socioeconomic status, 8 in smoking status, 26 in RV/TLC, 46 in DLco, 11 in PaO_2_, 10 in PaCO_2_, 30 in 6MWD, 2 in Charlson comorbidities and 8 in long-term oxygen therapy.

^+ ^One: only COPD; two or more: one or more comorbidities in addition to COPD.

^†^According to International Standard Classification of Occupations (ISCO).

**Table 2:** Health-related quality of life, psychological status, and limitations in activities of daily living of 337 COPD patients, according to ATS/ERS severity stages.

		Total n = 337	Mild n = 19	Moderate n = 161	Severe n = 130	Very severe n = 27	p*
**Health-related quality of life**							

SGRQ score (from 0 -no impairment- to 100)							

Symptoms	m (SD)	48.5 (17.6)	47.2 (13.8)	45.4 (16.5)	50.5 (18.4)	58.3 (18.7)	0.002^†^

Activity	m (SD)	47.2 (24.5)	27.5 (21.5)	39.8 (21.3)	54.4 (23.6)	71.2 (21.4)	< 0.001^‡^

Impact	m (SD)	26.5 (18.6)	13.4 (10.3)	22.0 (16.4)	30.5 (18.3)	43.2 (21.2)	< 0.001^‡^

Total	m (SD)	36.5 (17.8)	23.5 (11.5)	31.4 (15.1)	41.0 (17.5)	54.2 (18.8)	< 0.001^‡^

							

**Activities of daily living (n = 333)**							

Barthel Index (from 0 to 100)	median (P_25_-P_75_)	100 (100-100)	100 (100-100)	100 (100-100)	100 (100-100)	100 (97.5-100)	0.013^†^

Dependent (Barthel Index <100)	n (%)	30 (9.0)	0 (0)	10 (6.2)	14 (10.9)	6 (24.0)	0.020^†^

							

**Psychological status**							

HADS-anxiety score (from 0 to 21)	median (P_25_-P_75_)	5 (2-8)	6 (1-11)	5 (3-8)	4 (2-7)	5 (3-8)	0.898

HADS-depression score (from 0 to 21)	median (P_25_-P_75_)	3 (1-5.5)	2 (0-5)	3 (1-5)	3 (1-6)	4 (1-7)	0.533

HADS anxiety score ≥8	n (%)	91 (27.0)	8 (42.1)	46 (28.6)	29 (22.3)	8 (29.6)	0.267

HADS depression score ≥8	n (%)	46 (13.6)	4 (21.1)	20 (12.4)	16 (12.3)	6 (22.2)	0.349

HADS anxiety ≥8 and HADS depression ≥8	n (%)	30 (8.9)	4 (21.1)	13 (8.1)	9 (6.9)	4 (14.8)	0.122

HADS anxiety ≥8 and HADS depression <8	n (%)	61 (18.1)	4 (21.1)	33 (20.5)	20 (15.4)	4 (14.8)	0.664

HADS anxiety <8 and HADS depression ≥8	n (%)	16 (4.7)	0 (0)	7 (4.3)	7 (5.4)	2 (7.4)	0.732

COPD, Chronic obstructive pulmonary disease; ERS/ATS, European Respiratory Society/American Thoracic Society; SGRQ, St. George's Respiratory Questionnaire; HADS, Hospital Anxiety and Depression Scale.

* P-values in the table indicate that at least one of the severity groups differs from another severity group; ^† ^Separate analysis for each severity group showed that significant differences were observed between consecutive stages from moderate to very severe, without differences in the mild group; ^‡ ^Separate analysis for each severity group showed that significant differences were observed between all consecutive stages from mild to very severe.

In an adjusted multivariate model, lower FEV_1_, higher dyspnea scores, dependence, the presence of two or more Charlson comorbidities, and symptoms of anxiety, depression, or both, were related to a higher SGRQ Total score (or worse HRQoL) (Table 3). In addition to these factors, a lower BMI was associated with a higher SGRQ Impact score, while a lower 6MWD was independently associated with a higher SGRQ Activity score. Female gender was associated with a lower SGRQ Activity score. The SGRQ Symptoms domain exhibited a different pattern of determinants; being a current worker, having higher dyspnea scores, and both anxiety and depression were independently associated with higher scores. Notably, anxiety was more strongly associated with the Impact domain, while depression was more strongly associated with the Activity domain; the presence of both anxiety and depression was associated with the highest adjusted estimate in all SGRQ domains. The remaining potential confounders listed in the Methods section had no statistically significant association with any of the SGRQ domains. A multinomial logistic regression model revealed that current smoking, dependence (Barthel index < 100), and a higher socioeconomic status were independently related to anxiety and/or depression (Table 4).

**Table 3:** Adjusted association between psychological status and health-related quality of life (SGRQ).

	Total		Activity		Impact		Symptoms	
	β (95% CI)*	**p value**	β (95% CI)*	**p value**	β (95% CI)*	**p value**	β (95% CI)*	**p value**

**Psychological factors:**								
**Anxiety only**	6.02 (2.88, 9.16)	< 0.001	4.23 (-0.07, 8.52)	0.054	8.71 (4.93, 12.49)	< 0.001	4.07 (-0.42, 8.56)	0.076
**Depression only**	6.29 (0.55, 12.03)	0.032	11.34 (2.92, 19.77)	0.008	5.90 (-1.03, 12.82)	0.095	4.25 (-3.85, 12.34)	0.303
**Anxiety and depression**	17.06 (12.50, 21.62)	< 0.001	17.73 (11.24, 24.22)	< 0.001	18.35 (12.79, 23.91)	< 0.001	11.49 (5.36, 17.62)	< 0.001

**Gender: females**	-	-	-7.84 (-14.67, -1.01)	0.025	-	-	-	-

**Working status: current workers**	**-**	-	-	-	-	-	8.03 (3.43, 12.64)	0.001

**ATS/ERS severity stages:**								
**Mild**	reference		reference		reference			
**Moderate**	5.25 (-0.09, 10.58)	0.054	5.72 (-1.58, 13.01)	0.124	7.34 (0.91, 13.77)	0.025	-	-
**Severe**	11.25 (5.73, 16.76)	< 0.001	14.49 (6.89, 22.09)	< 0.001	12.93 (6.28, 19.57)	< 0.001		
**Very severe**	16.84 (9.78, 23.90)	< 0.001	18.25 (8.35, 28.14)	< 0.001	18.51 (9.92, 27.10)	< 0.001		

**6MWD (m)**	-	-	-0.02 (-0.04, -0.00)	0.033	-	-	-	-

**Dyspnea (score from 0 to 5)**	6.87 (5.90, 7.85)	< 0.001	9.87 (8.46, 11.28)	< 0.001	5.54 (4.37, 6.72)	< 0.001	4.36 (3.09, 5.64)	< 0.001

**BMI: <20 (kg/m^2^)**	-	-	-	-	9.93 (0.90, 18.96)	0.031	-	-

**Barthel Index: dependent^†^**	6.67 (2.18, 11.15)	0.003	10.73 (3.14, 18.31)	0.006	5.76 (0.35, 11.17)	0.037	-	-

**Charlson Comorbidities: ≥2**	3.70 (1.26, 6.15)	0.003	4.94 (1.53, 8.35)	0.005	3.91 (0.96, 6.86)	0.010	-	-

**Adjusted R^2^**	0.61		0.65		0.49		0.20	

CI: confidence interval

* Each column is a single multivariate lineal regression model including as covariates the variables that show a coefficient in each column. Beta-coefficients are expressed in points of the SGRQ score per (i) each unit of the continuous covariates in the model, or (ii) a change with respect to reference category in categorical covariates.

† Dependent defined as Barthel Index < 100

Age, socioeconomic status, level of education, marital status, smoking status, DLco, RV/TLC, PaO_2 _and PaCO_2_, were tested as potential confounders and finally not included because they were not independently related to the outcome, nor modified estimates for the remaining variables.

**Table 4:** Variables associated with Anxiety and/or Depression in patients with COPD (multinomial (polytomous) logistic regression)*

	Anxiety		Depression		Anxiety and Depression	
	
	OR (95% CI)	p	OR (95% CI)	p	OR (95% CI)	p
**Socioeconomic status: I-III^†^**	-	-	5.51 (1.73, 17.53)	0.004	-	-

**Smoking status: current smokers**	2.52 (1.36, 4.69)	0.004	5.70 (1.73, 18.77)	0.004	3.40 (1.33, 8.70)	0.010

**Barthel Index: dependent**	-	-	17.34 (4.20, 71.63)	< 0.001	9.93 (3.18, 30.99)	< 0.001

CI: confidence interval; OR: odds ratio

*The whole table is a single model with neither anxiety nor depression as the reference group. Other relevant potential confounders (see Methods), were also tested but not included in the final model because they were not independently related to the outcome, nor modified estimates for the remaining variables.

^† ^According to International Standard Classification of Occupations (ISCO); I-III: high socioeconomic status.

**Figure 1:**
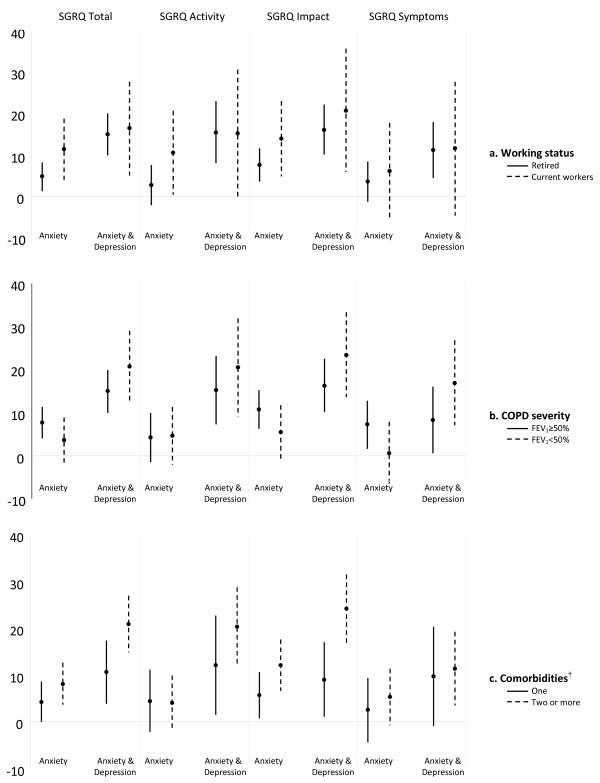
**Association between psychological status and health-related quality of life in 337 stable COPD patients according to working status (a), COPD severity (b), and comorbidities (c)**. Multivariate models adjusted by covariates in Table 3. The dots represent the regression coefficients and the vertical bars indicate the 95% confidence intervals. These estimates are interpreted as the change in SGRQ score (points) with respect to the reference group (subjects with neither anxiety nor depression (horizontal line)). Subjects with depression only were not included due to small sample size. † Chronic diseases from the Charlson index. One: only COPD; two or more: one or more in addition to COPD.

Effect modification by working status, severity of COPD, and comorbidity is shown in Figure 1. The effect of work status on the association between "anxiety alone" and HRQoL was stronger in current workers than in those who were retired. Among current workers, individuals with anxiety had SGRQ scores that were 11, 10, 14 and 6 points higher than the reference group (neither anxiety nor depression) for Total, Activity, Impact and Symptoms, respectively. Among retired, individuals with anxiety had SGRQ scores that were 5, 2, 8 and 4 points higher than the reference group for Total, Activity, Impact and Symptoms, respectively. Thus, the differences observed in effect estimates were clinically relevant (higher than 4 points) for the SGRQ Total, Activity, and Impact domains (Figure 1a). Regarding effect modification by severity of COPD (Figure 1b), "anxiety alone" showed a greater effect on the Total, Impact, and Symptoms scores in those with mild-to-moderate COPD than in those with severe-to-very severe COPD. Among patients with mild-to-moderate COPD, patients with anxiety and depression had SGRQ scores that were 14, 15, 16 and 8 points higher than the reference group, for Total, Activity, Impact and Symptoms, respectively. However, among COPD patients with severe-to-very severe COPD, patients with anxiety and depression had SGRQ scores that were 20, 20, 23 and 17 points higher than the reference group for Total, Activity, Impact and Symptoms, respectively. Overall, COPD patients with anxiety and depression reported worse HRQoL than those with neither anxiety nor depression regardless of severity stage of COPD, but the effect of anxiety and depression on HRQoL was stronger among COPD patients with severe-to-very severe disease than among those with mild-to-moderate COPD. Similarly, the magnitude of the association between "both anxiety and depression" and HRQoL (Total, Activity and Impact domains) was stronger in COPD patients with two or more Charlson comorbidities than in patients with one comorbidity; this association was especially notable in the Impact domain (Figure 1c). Other potential effect modifiers, including gender, age, marital status, smoking, level of education, and socioeconomic status, were tested. Relevant differences in effect estimates of the association between "both anxiety and depression" and HRQoL were observed (1) among COPD patients under 69, compared to older subjects (21 *vs *15 points for Impact domain); (2) among current smokers compared to former smokers (19 *vs *14 points for Total, 21 *vs *15 for Impact, 19 *vs *15 for Activity, and 13 *vs *9 for Symptoms); and (3) among subjects with high rather than low socioeconomic status (23 *vs *18 and 25 *vs *19 for Total and Impact domains, respectively).

## Discussion

In the present study, we found that anxiety, depression, or the combination of both were significantly associated with poor HRQoL (measured by SGRQ) in a large sample of stable COPD patients, even after adjustment for potential confounders. This association was modified by socio-demographic factors (age, work status, and socioeconomic status), smoking status, severity of COPD, and the presence of comorbidities.

Our results are consistent with and complement those of previous studies that identified a relationship between psychological impairment and reduced HRQoL in COPD patients [10-12]. While most previous studies have focused on the effects of depression with little attention to anxiety [11,12,25], we examined them separately allowing to identify different patterns, such as strong associations between anxiety and the Impact domain, or between depression and the Activity domain. We have investigated this aspect in a sample of stable COPD patients who have a broad spectrum of disease severity. Additionally, the wide characterisation of our sample has allowed us to adjust for potential confounders when appropriate, including socio-demographic characteristics, smoking, clinical-functional characteristics, exercise capacity, nutritional status, or comorbidities; this adjustment reduces the possibility that the results could be attributed to factors other than psychological factors.

A clinically relevant finding of this study is the greater association between anxiety and poor HRQoL among current workers than retired patients in all SGRQ domains. COPD is often related to work disability and to the perception of limitations in work capacity [26,27]. In the present study, current workers had higher Symptoms scores after adjustment for age, smoking status, and severity of COPD; however, they did not show higher psychological impairment. Altogether, our results suggest that the presence of anxiety in current workers could negatively influence their perceptions of work limitations and their process of adapting to these limitations. A potential clinical implication of these results includes the need to assess and eventually treat psychological impairment in COPD patients who are active workers. To which extent the early retirement in workers with COPD could result in a benefit for HRQoL in these patients goes beyond the current study, and requires further specific longitudinal studies.

The pattern of effect modification by severity of COPD is of particular interest. Patients with mild-to-moderate COPD, in comparison with those with severe-to-very severe COPD, showed a stronger association between having "anxiety alone" and poor HRQoL. A previous study [13] conducted on patients with mild-to-severe COPD, reported that anxiety was associated with HRQoL only in the subset of patients with FEV_1 _below 60% of the predicted value. Compared to previous research, our results show a high prevalence of anxiety, even in patients with less advanced COPD [28]. This fact, together with the substantial impact of anxiety on the HRQoL of patients with mild-to-moderate COPD, is clinically relevant and reinforces current recommendations for screening for psychiatric comorbidities in patients who have been diagnosed with COPD [29]. In contrast, we have found that when anxiety and depression appear together, their influence on HRQoL is greater in patients with severe-to-very severe COPD than in those with mild-to-moderate COPD. In fact, the prevalence of depression in our study was lower than in previous studies [10,11,28] and was not related to COPD severity; this finding contradicts the results of previous research [12]. Such differences could be related to the fact that we selected patients at an earlier stage of the disease than previous studies. The different profile of associations between anxiety and HRQoL and depression and HRQoL, according to COPD severity, needs to be examined further.

The presence of comorbidities was associated with reduced HRQoL, as found in other studies [4,5]. It is interesting to note that the impact on HRQoL was higher when patients suffered from both anxiety and depression and had other comorbidities in addition to COPD. This finding is in agreement with the current approach that COPD patients should benefit form integrated care programs, centered on the patient rather than the disease, that are currently promoted by the WHO for chronic diseased patients [30].

Surprisingly, we found a greater influence of having "both anxiety and depression" on the SGRQ Impact domain in younger (less than 69 years) patients than in older patients. Previous studies have described a higher prevalence of anxiety and depression in younger COPD patients [31,32], which was not observed in our data. The observed effect modification by age in our study is most likely explained by working status because younger subjects are more likely to be current workers.

With respect to the different domains of the SGRQ, it is remarkable to note that patients showed lower scores in the Impact domain than in the Symptoms or Activity domains; the Impact domain was also strongly associated with anxiety (alone or with depression). The origin of the Impact domain, covering psychological disturbances resulting from respiratory disease, partly explains these findings. A *posthoc *analysis excluding psychological items from SGRQ resulted in still clinically relevant and statistically significant associations between anxiety, depression, or both, and the Impact domain, suggesting that psychological status plays an important role also in the social function of COPD patients. Interestingly, the associations between anxiety, alone or with depression, and the Impacts domain showed larger variations after stratification by most potential effect modifiers than the corresponding associations with Symptoms or Activity domains.

Some limitations of our study should be mentioned. First, the cross-sectional design of the study does not allow us to determine a causal relationship between psychological factors and HRQoL. Second, although the HADS has been used in other large studies of patients with COPD [10,11,13,22], it is a screening tool rather than a diagnostic instrument. The diagnosis of specific anxiety or depressive disorders (DSM-IV) should be made by a qualified mental health professional through a structured clinical interview [8]. The use of a screening tool may have resulted in the inclusion of healthy subjects in the anxiety or depression groups, which would not detract from our results. Finally, the small number of patients with depression alone, as well as the small number of females, limits our conclusions with regard to these specific subgroups.

An important strength of our study, in addition to the large sample size and extensive control for appropriate confounders, is the recruitment of patients at their first admission. This criterion allows us to measure HRQoL, psychological status, and covariates without confusion from previous admissions.

## Conclusions

In summary, this study examines a relatively large sample of stable patients with mild to very severe COPD. The study identified a significant association between anxiety, depression, or both and reduced HRQoL and found that the magnitude of this association was modified by working status, COPD severity, and the presence of comorbidities. A better understanding of the influence of psychological status on HRQoL at earlier stages of COPD, including the identification of patients most vulnerable to psychological impairment, is required. This improved understanding could enable the application of specific strategies in both clinical practice and public health that seek to prevent further disability associated with COPD.

## List of abbreviations

COPD: Chronic Obstructive Pulmonary Disease; HRQoL: Health-related quality of life; SGRQ: St. George's Respiratory Questionnaire; SD: Standard deviation; PAC-COPD: Phenotype and Course of COPD; HADS: Hospital Anxiety and Depression Scale; MMRC: Modified Medical Research Council; ISCO: International Standard Classification of Occupations; BMI: body mass index; FEV_1_: forced expiratory volume in one second; FEV_1_/FVC: forced expiratory volume in one second/forced vital capacity; DLco: diffusing capacity for carbon monoxide; RV/TLC: residual volume/total lung capacity; PaO_2_: partial pressure of oxygen in arterial blood; PaCO_2_: partial pressure of carbon dioxide in arterial blood; ANOVA: Analysis Of Variance; CI: Confidence Interval; OR: Odds ratio; 6MWD: six-minute walking distance; ERS: European Respiratory Society; ATS: American Thoracic Society.

## Competing interests

The authors declare that they have no competing interests.

## Authors' contributions

All authors have contributed to (1) the conception and design of the study; (2) analysis and interpretation of data; and (3) writing the article or revising it critically for important intellectual content. EB, JG-A, MB, and IS performed the statistical analysis and interpreted the results; EB prepared the first draft of the paper; EB and JG-A had full access to all of the data in the study and take responsibility for the integrity of the data and the accuracy of the data analysis. All authors read and approved the final manuscript.
